# Pyrosequencing-Based Analysis of the Microbiome Associated with the Horn Fly, *Haematobia irritans*

**DOI:** 10.1371/journal.pone.0044390

**Published:** 2012-09-24

**Authors:** Azhahianambi Palavesam, Felix D. Guerrero, Andrew M. Heekin, Ju Wang, Scot E. Dowd, Yan Sun, Lane D. Foil, Adalberto A. Pérez de León

**Affiliations:** 1 USDA-ARS Knipling-Bushland US Livestock Insects Research Laboratory, Kerrville, Texas, United States of America; 2 Institute for Bioscience and Biotechnology Research, University of Maryland, College Park, Maryland, United States of America; 3 Molecular Research LP, Shallowater, Texas, United States of America; 4 Research and Testing Laboratory, Lubbock, Texas, United States of America; 5 Department of Entomology, Louisiana State University, Baton Rouge, Louisiana, United States of America; 6 Department of Veterinary Parasitology, Madras Veterinary College, Tamil Nadu Veterinary and Animal Sciences University, Chennai, Tamil Nadu, India; Centro de Pesquisas René Rachou, Brazil

## Abstract

The horn fly, *Haematobia irritans*, is one of the most economically important pests of cattle. Insecticides have been a major element of horn fly management programs. Growing concerns with insecticide resistance, insecticide residues on farm products, and non-availability of new generation insecticides, are serious issues for the livestock industry. Alternative horn fly control methods offer the promise to decrease the use of insecticides and reduce the amount of insecticide residues on livestock products and give an impetus to the organic livestock farming segment. The horn fly, an obligatory blood feeder, requires the help of microflora to supply additional nutrients and metabolize the blood meal. Recent advancements in DNA sequencing methodologies enable researchers to examine the microflora diversity independent of culture methods. We used the bacterial 16S tag-encoded FLX-titanium amplicon pyrosequencing (bTEFAP) method to carry out the classification analysis of bacterial flora in adult female and male horn flies and horn fly eggs. The bTEFAP method identified 16S rDNA sequences in our samples which allowed the identification of various prokaryotic taxa associated with the life stage examined. This is the first comprehensive report of bacterial flora associated with the horn fly using a culture-independent method. Several rumen, environmental, symbiotic and pathogenic bacteria associated with the horn fly were identified and quantified. This is the first report of the presence of *Wolbachia* in horn flies of USA origin and is the first report of the presence of *Rikenella* in an obligatory blood feeding insect.

## Introduction

The horn fly, *Haematobia irritans*, is a hematophagous external parasite of cattle. Both male and female horn flies spend their entire life on the host and feed on blood, 24–38 times/day, using their piercing and sucking proboscis [1]. Economic losses in cattle production systems are the result of extreme irritation and annoyance caused by horn fly biting and blood sucking, which cause reduction in feed efficiency, daily weight gain and milk production [2]. It has been estimated that cattle producers in the U.S.A. lose nearly one billion dollars annually due to the effect of horn fly infestations [3], [4]. Substantial losses are also incurred in Brazil, Argentina and Chile [5], [6].

Horn flies vector the filarial nematode *Stephanofilaria stilesi* in North America [7] and in Brazil they were shown to be a phoretic vector of the human bot fly *Dermatobia hominis* [8]. Moreover, horn flies act as mechanical vectors of different pathogens that cause disease in cattle [9]–[15]. Chemical control has been a major element of horn fly management programs. However, the evolution of resistance to many insecticides highlights the need to develop alternative control methods [16]. Development of effective non-chemical alternative methods to control the horn fly could reduce the use of insecticides on animals, reduce insecticide residues on livestock products, and boost the organic livestock farming segment. The development of such management strategies necessitates continued research on horn fly biology. A comprehensive understanding of the biology of any insect requires study in an ecological context with microorganisms as an important component of the study system [17].

Bacteria are ubiquitous organisms and represent a major part of the insect-associated microbiome. Bacteria-insect relationships date back millions of years. For example, *Bacillus* DNA was amplified from 25- to 40-million-year-old bees preserved in amber [18]. The association of prokaryotes with eukaryotic biological systems is reflected in various relationships that are characterized by the type of host interaction that evolved. Depending on the relationship, bacteria can be a parasitic, symbiotic, or commensal to its host insect. Comprehensive studies of an insect's microbiome by classical culture-based methods are difficult. Several molecular-based techniques have revealed that more than 99% of microorganism existing in nature cannot be isolated and maintained in pure culture [19]. Recent advancements in DNA sequencing technology and bioinformatics offer expedient and efficient tools to analyze bacterial communities of insects, avoiding the need for intensive culture-based techniques, as well as allowing the identification of bacterial species that are not amenable to conventional culture methods [20]–[22]. In the present study, bacterial 16S tag-encoded FLX-titanium amplicon pyrosequencing (bTEFAP) [21], [22] was used to compile a broad survey of the bacterial community of *H. irritans*, identifying and quantifying the bacteriome of three life stages: adult male, adult female, and eggs.

## Results

### 16S tag-encoded FLX-titanium amplicon pyrosequencing (bTEFAP)

A total of 98,526 sequences were generated and a total of 66,243 sequences from 9 different biological samples were used for analyses. All failed sequence reads, low quality sequence ends and tags were removed and sequences were depleted of any non-bacterial ribosomal sequences and chimeras using custom software described previously [21] and the black box chimera check software B2C2. An average of 7,360 DNA sequences (range 2527–11,096) of >350 bp (average length 450 bp) were used per sample for analyses after all quality control and screening steps were performed.

### Operational Taxonomic Unit (OTU) determination

Indices of bacterial diversity and abundance, based on Operational Taxonomic Unit (OTU), were estimated through rarefaction curve analysis. Rarefaction and Richards maximum predicted curve modeling indicated that >97% of OTUs at 3% divergence were achieved for each sample [23], which suggests adequate depth of coverage. The estimated OTUs through rarefaction analysis were 261, 177 and 93 for horn fly eggs, adult male horn fly and adult female horn fly, respectively (Table S1). Rarefaction curves were rendered for all nine samples (Figure S1) at the 0.03 divergence level. The rarefaction curves imply a depth of coverage of approximately 10000 sequences/sample for the female and egg samples. This depth of coverage allows inference of the frequencies of species at 0.1% relative abundance with reasonable accuracy. For example, 10 sequences are expected per taxon with 0.1% relative abundance at this depth. The male horn fly samples, however, were only sampled to about one third of the depths of either the female or egg samples.

### Bacteriome of the horn fly

**Figure 1:**
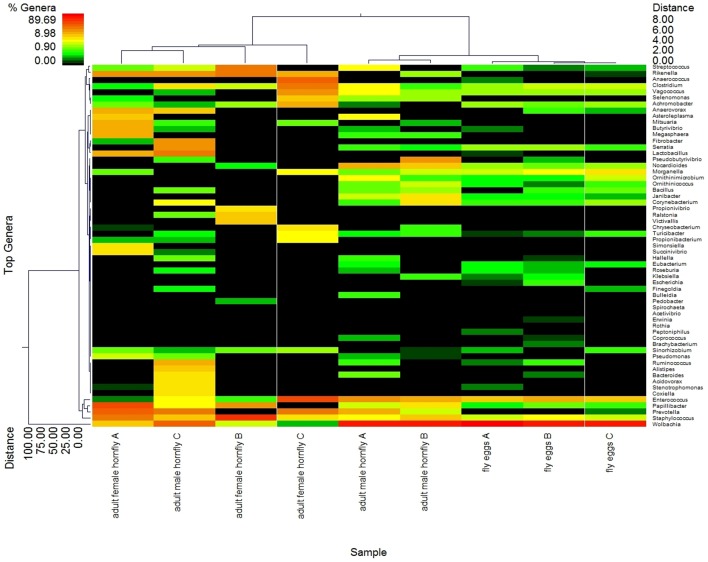
Heat map depicting bacterial diversity and relative abundance in life stages from the horn fly. Letters (A–C) used to identify individual life stage replicates. Double hierarchical dendogram shows different bacteria distribution between genera based on complete linkage clustering, and Manhattan distance methods with no scaling. Dendrogram linkages and distance of the bacterial genus or trace back groups are not phylogenetic, but based upon relative abundance of the genus within the samples. Traceback means bacterial classifications were based upon the percent identity of the sample sequence to known sequences, the percent divergence was then used to adjust identifications to the taxonomic level with the highest degree of confidence (e.g. a percent divergence <3% can be expected to provide confidence at the species level, >3% but <5% at the genera level, etc). Classifications were compiled after traceback. Legend and scale shown in upper left corner of the figure represent colors in heat map associated with the relative percentage of each traceback grouping of bacteria (cluster of variables in Y-axis) within each fly sample (X-axis clustering). Fly samples along the X-axis with Manhattan distances are indicated by branch length and associated with the scale located at the upper right corner of the figure. Bacterial genera along the Y-axis are also clustered according to Manhattan distances; the respective scale is indicated in the figure's lower left corner.

From the heat map, it is evident that the bacterial diversity and relative abundance patterns for the egg sample replicates most closely resemble one another as compared to those for the adult male and adult female replicates (Fig. 1). Bacterial diversity and relative abundance between one of the adult male replicates (sample C) and female sample A, are somewhat similar because this male sample contains a lesser abundance of *Wolbachia* than male sample replicates A and B. In other aspects of the overall heat map abundance pattern, male sample C does not closely resemble the three female replicate samples. Bacterial 16S rDNA-based pyrosequencing allowed us to identify and assess the relative abundance of the taxonomic levels of bacteria in male and female horn flies and horn fly eggs (Fig. 1). BLASTn searches identified a total of 25 unique classes with 363 genera including 76 taxa at the species level [24], [25]. The combined species and genus data is listed in Table S1. The mean normalized abundance of the majority of the genera and species (87.5%) was less than 0.1%, which could be attributed to the extremely high level of *Wolbachia* in the male fly and egg samples. A cut-off filter based on abundance level ≥0.1% and prevalence of taxa ≥66% within each class respectively (or a prevalence ≥55% across all nine samples) was used to select the predominant taxa. Among the 363 OTUs identified, only 50 met these criteria. With these criteria, 9 OTUs were identified at the species level, *Prevotella buccalis*, *Janibacter anopheles*, *Clostridium lituseburense*, *Morganella morganii*, *Staphylococcus saprophyticus*, *Serratia marcescens*, *Staphylococcus hyicus*, *Staphylococcus sciuri* and *Staphylococcus aureus*.

### Diversity and abundance of the bacterial community in the horn fly

**Figure 2:**
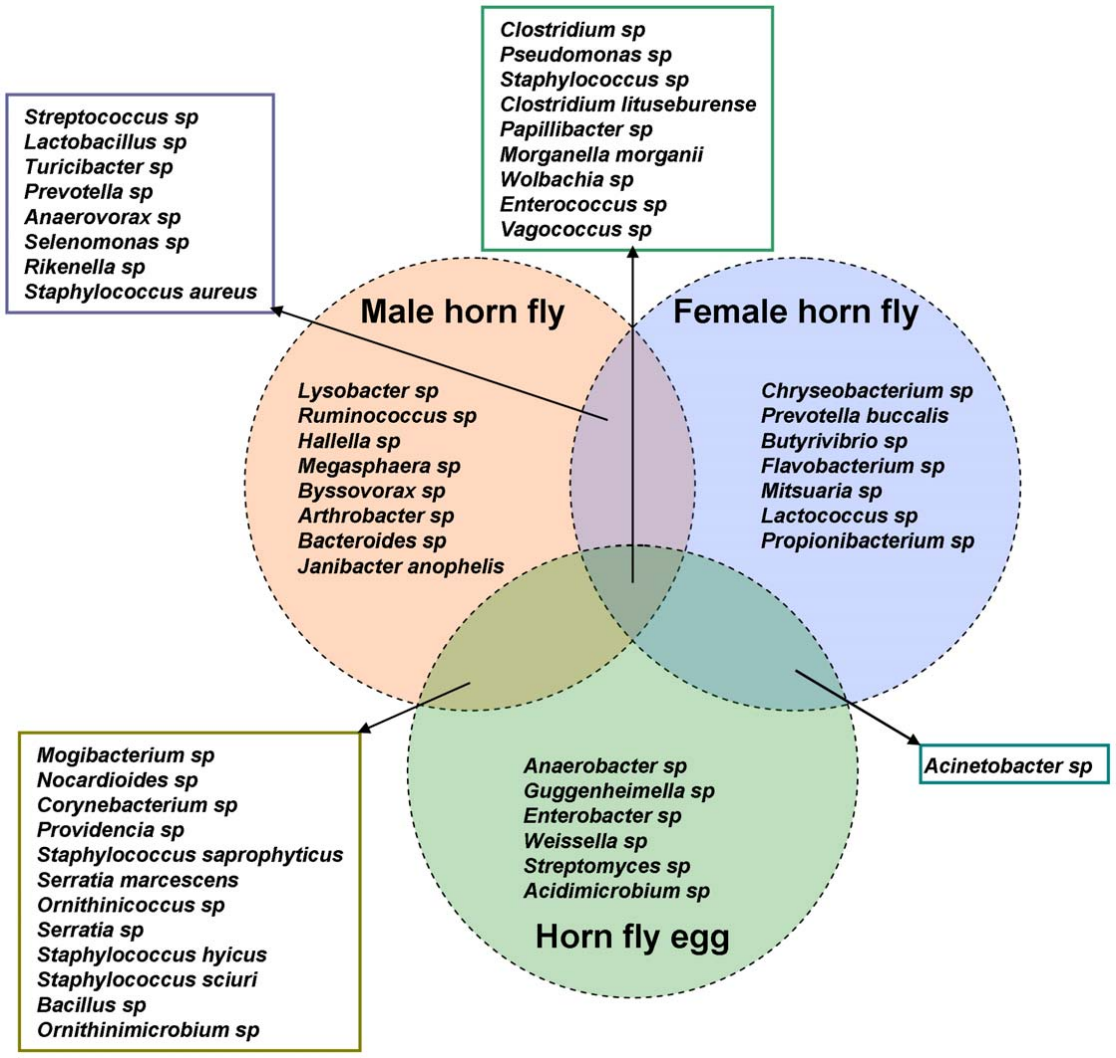
Distribution and overlapping of predominant taxa in adult female horn fly, adult male horn fly and horn fly egg after filtering with a cut-off filter based on abundance level ≥0.1% and prevalence of taxa ≥66% within each class (or a prevalence ≥55% across all nine samples).

**Figure 3:**
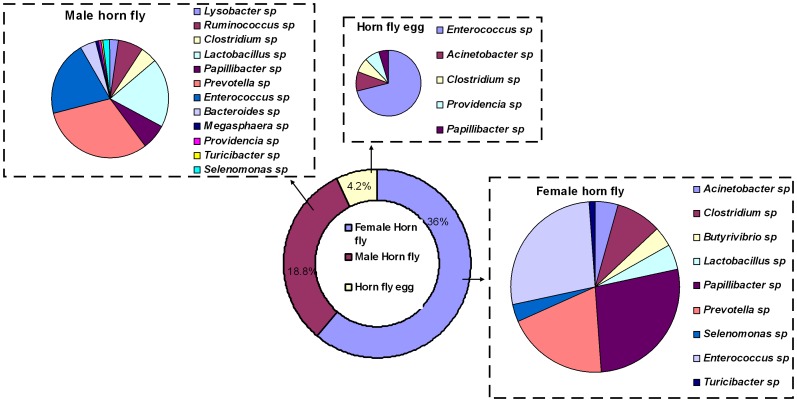
A pie diagram showing the abundance and diversity of cattle rumen and gastro-intestinal bacteria in adult female, male horn fly and horn fly egg.

After applying the cut-off filter described above, the total number of OTUs detected in adult male, adult female and horn fly eggs were 37, 25 and 28, respectively (Table 1), while the number of OTUs unique to each sample type were 8, 7 and 6, respectively (Fig. 2). The number of bacterial genera/species shared between adult male and female horn fly, adult male and horn fly eggs and adult female and horn fly eggs were 17, 21 and 10, respectively (Table 2). *Wolbachia* was the most abundant bacteria detected in the horn fly. It comprised 1%, 52.4% and 86% abundances in adult female, adult male, and eggs, respectively (Table S1). Rumen and gastrointestinal bacteria of cattle [26] were the second most abundant bacteria found in horn fly bacterial flora after *Wolbachia* with abundance levels of 36%, 18.8% and 4.2% in adult female, adult male, and eggs, respectively (Table S1) (Fig. 3). *Clostridium*, *Pseudomonas*, *Staphylococcus*, *Clostridium lituseburense*, *Papillibacter*, *Morganella morganii*, *Wolbachia*, *Enterococcus*, *and Vagococcus* were the 9 OTUs found among all classes (Fig. 2).

**Table 1:** List of predominant bacteria in adult male horn fly, adult female horn fly and horn fly egg after filtering with a cut-off filter based on abundance level ≥0.1% and prevalence of taxa ≥66% within each class (or a prevalence ≥55% across all nine samples).

Adult male horn fly	Adult female horn fly	Horn fly egg
*1. Lysobacter sp*	*1. Acinetobacter sp*	*1. Morganella morganii*
*2. Ruminococcus sp*	*2. Chryseobacterium sp*	*2. Weissella sp*
*3. Clostridium sp*	*3. Clostridium sp*	*3. Wolbachia sp*
*4. Nocardioides sp*	*4. Streptococcus sp*	*4. Enterococcus sp*
*5. Corynebacterium sp*	*5. Prevotella buccalis*	*5. Acinetobacter sp*
*6. Lactobacillus sp*	*6. Butyrivibrio sp*	*6. Mogibacterium sp*
*7. Staphylococcus sp*	*7. Lactobacillus sp*	*7. Clostridium sp*
*8. Papillibacter sp*	*8. Staphylococcus sp*	*8. Nocardioides sp*
*9. Prevotella sp*	*9. Pseudomonas sp*	*9. Anaerobacter sp*
*10. Staphylococcus aureus*	*10. Papillibacter sp*	*10. Corynebacterium sp*
*11. Serratia marcescens*	*11. Prevotella sp*	*11. Guggenheimella sp*
*12. Anaerovorax sp*	*12. Mitsuaria sp*	*12. Staphylococcus sp*
*13. Wolbachia sp*	*13. Staphylococcus aureus*	*13. Pseudomonas sp*
*14. Rikenella sp*	*14. Anaerovorax sp*	*14. Providencia sp*
*15. Enterococcus sp*	*15. Wolbachia sp*	*15. Clostridium lituseburense*
*16. Janibacter anophelis*	*16. Selenomonas sp*	*16. Papillibacter sp*
*17. Serratia sp*	*17. Rikenella sp*	*17. Enterobacter sp*
*18. Ornithinimicrobium sp*	*18. Enterococcus sp*	*18. Staphylococcus saprophyticus*
*19. Vagococcus sp*	*19. Vagococcus sp*	*19. Serratia marcescens*
*20. Bacteroides sp*	*20. Flavobacterium sp*	*20. Staphylococcus hyicus*
*21. Mogibacterium sp*	*21. Turicibacter sp*	*21. Ornithinicoccus sp*
*22. Hallella sp*	*22. Clostridium lituseburense*	*22. Serratia sp*
*23. Streptococcus sp*	*23. Morganella morganii*	*23. Staphylococcus sciuri*
*24. Megasphaera sp*	*24. Propionibacterium sp*	*24. Streptomyces sp*
*25. Pseudomonas sp*	*25. Lactococcus sp*	*25. Bacillus sp*
*26. Providencia sp*		*26. Ornithinimicrobium sp*
*27. Turicibacter sp*		*27. Acidimicrobium sp*
*28. Clostridium lituseburense*		*28. Vagococcus sp*
*29. Morganella morganii*		
*30. Staphylococcus saprophyticus*		
*31. Selenomonas sp*		
*32. Staphylococcus hyicus*		
*33. Arthrobacter sp*		
*34. Ornithinicoccus sp*		
*35. Staphylococcus sciuri*		
*36. Bacillus sp*		
*37. Byssovorax sp*		

**Table 2:** List of predominant bacteria overlapping of between adult female horn fly, adult male horn fly and horn fly egg after filtering with a cut-off filter based on abundance level ≥0.1% and prevalence of taxa ≥66% within each class (or a prevalence ≥55% across all nine samples).

Bacteria shared between adult male and female horn fly	Bacteria shared between adult male and horn fly egg	Bacteria shared between adult female and horn fly egg
*1. Streptococcus sp*	*1. Mogibacterium sp*	*1. Clostridium sp*
*2. Lactobacillus sp*	*2. Nocardioides sp*	*2. Pseudomonas sp*
*3. Turicibacter sp*	*3. Corynebacterium sp*	*3. Staphylococcus sp*
*4. Prevotella sp*	*4. Providencia sp*	*4. Clostridium lituseburense*
*5. Staphylococcus aureus*	*5. Staphylococcus saprophyticus*	*5. Papillibacter sp*
*6. Selenomonas sp*	*6. Serratia marcescens*	*6. Morganella morganii*
*7. Rikenella sp*	*7. Ornithinicoccus sp*	*7. Wolbachia sp*
*8. Anaerovorax sp*	*8. Serratia sp*	*8. Enterococcus sp*
*9. Clostridium sp*	*9. Staphylococcus hyicus*	*9. Vagococcus sp*
*10. Pseudomonas sp*	*10. Staphylococcus sciuri*	*10. Acinetobacter sp*
*11. Staphylococcus sp*	*11. Bacillus sp*	
*12. Clostridium lituseburense*	*12. Ornithinimicrobium sp*	
*13. Papillibacter sp*	*13. Clostridium sp*	
*14. Morganella morganii*	*14. Pseudomonas sp*	
*15. Wolbachia sp*	*15. Staphylococcus sp*	
*16. Enterococcus sp*	*16. Clostridium lituseburense*	
*17. Vagococcus sp*	*17. Papillibacter sp*	
	*18. Morganella morganii*	
	*19. Wolbachia sp*	
	*20. Enterococcus sp*	
	*21. Vagococcus sp*	

**Figure 4:**
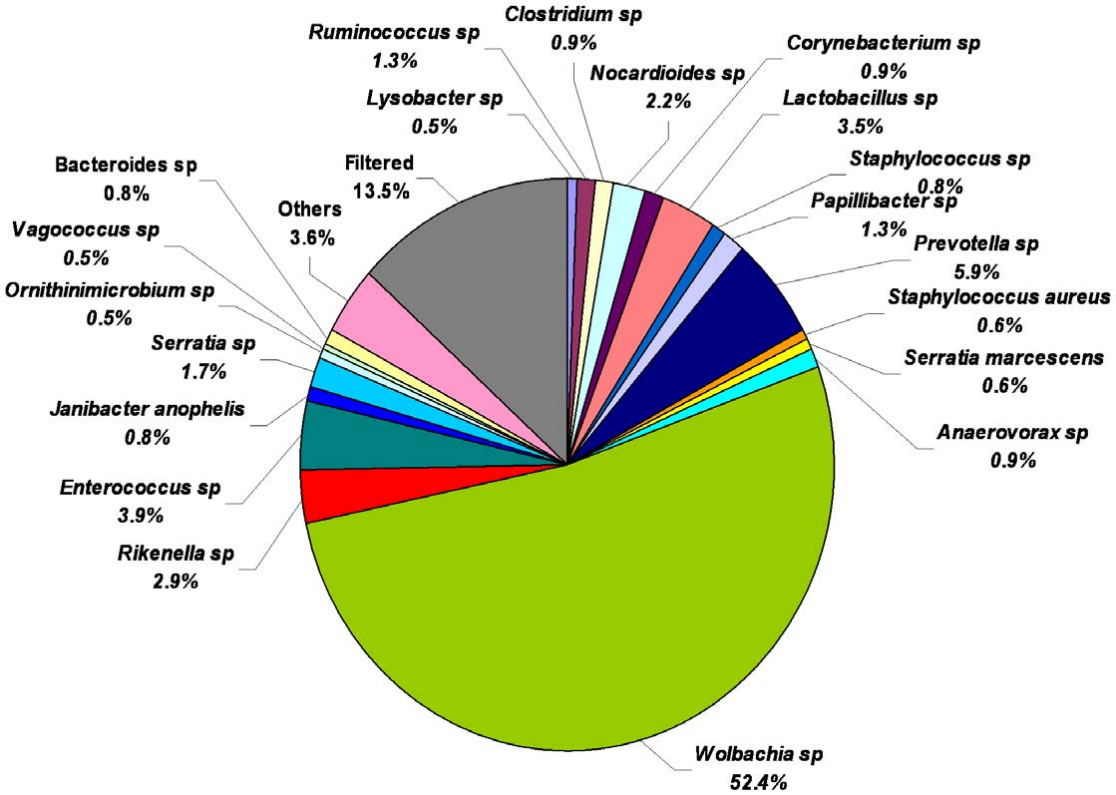
Relative abundance of bacterial genera/species in adult male horn fly after applying the cut-off filter of abundance level ≥0.1% and prevalence of taxa ≥66% within each class respectively or a prevalence ≥55% across all nine samples. Values are mean percentages (n = 3).

Among the 37 OTUs identified in males included, *Janibacter anopheles*, *Clostridium lituseburense*, *Morganella morganii*, *Staphylococcus saprophyticus*, *Serratia marcescens*, *Staphylococcus hyicus*, *Staphylococcus sciuri*, and *Staphylococcus aureus* were the bacteria identified to the level of species. *Wolbachia* was the most abundant (52.4%) bacterial species in adult male horn fly. *Prevotella* (5.9%), *Enterococcus* (3.9%), *Lactobacillus* (3.5%), *Rikenella* (2.9%), *Nocardioides* (2.2%), *Serratia* (1.7%), *Ruminococcus* (1.3%) and *Papillibacter* (1.3%) were the other major OTUs associated with adult male horn flies (Fig. 4).

**Figure 5:**
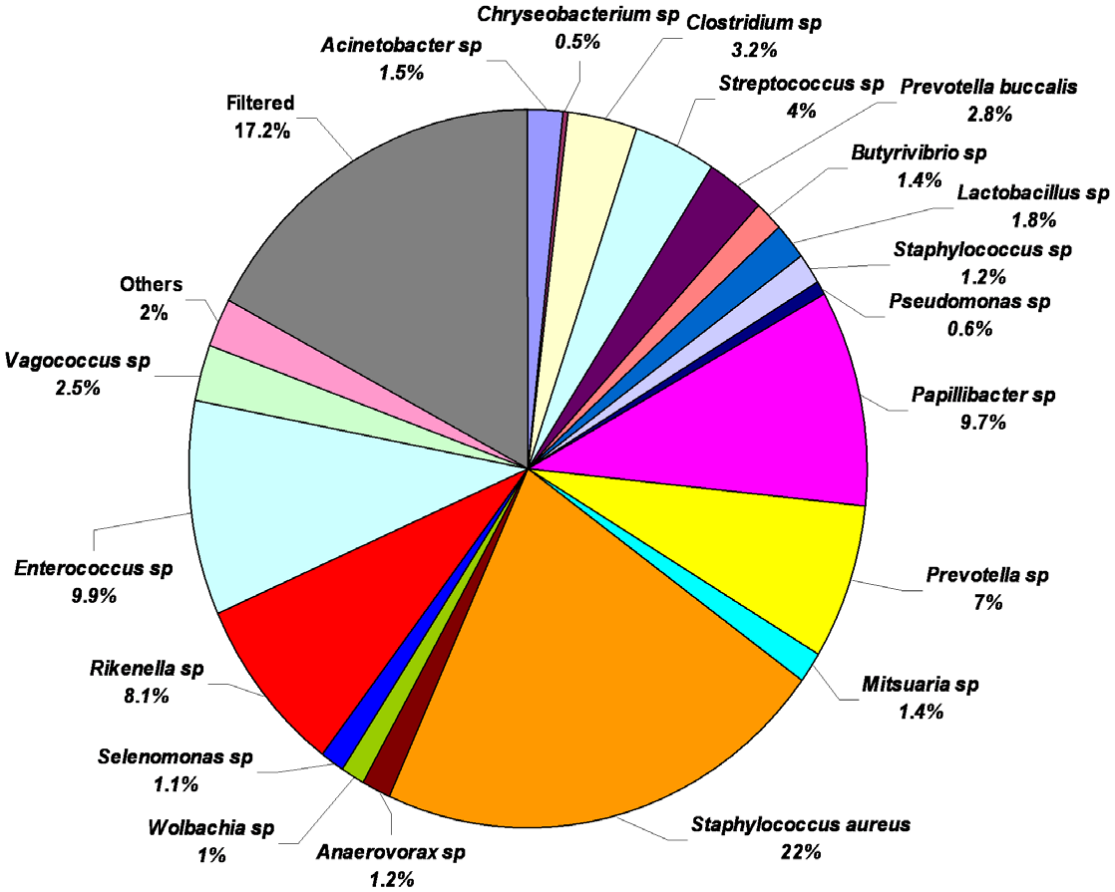
Relative abundance of bacterial genera/species in adult female horn fly after applying the cut-off filter of abundance level ≥0.1% and prevalence of taxa ≥66% within each class respectively or a prevalence ≥55% across all nine samples. Values are mean percentages (n = 3).

Among the 25 OTUs identified in females, *Prevotella buccalis*, *Clostridium lituseburense*, *Morganella morganii* and *Staphylococcus aureus* were the bacteria identified at the level of species. Figure 5 shows that *Staphylococcus aureus* was the most abundant bacteria in adult female horn fly (22%), followed by *Enterococcus* (9.9%), *Papillibacter* (9.7%), *Rikenella* (8.1%), *Prevotella* (7%), *Streptococcus* (4%), *Clostridium* (3.2%), *Prevotella buccalis* (2.8%), *Vagococcus* (2.5%), *Lactobacillus* (1.8%), *Acinetobacter* (1.5%), *Butyrivibrio* (1.4%), *Mitsuaria* (1.4%), *Staphylococcus* (1.2%), *Anaerovorax* (1.2%), *Selenomonas* (1.1%) and *Wolbachia* (1%).

**Figure 6:**
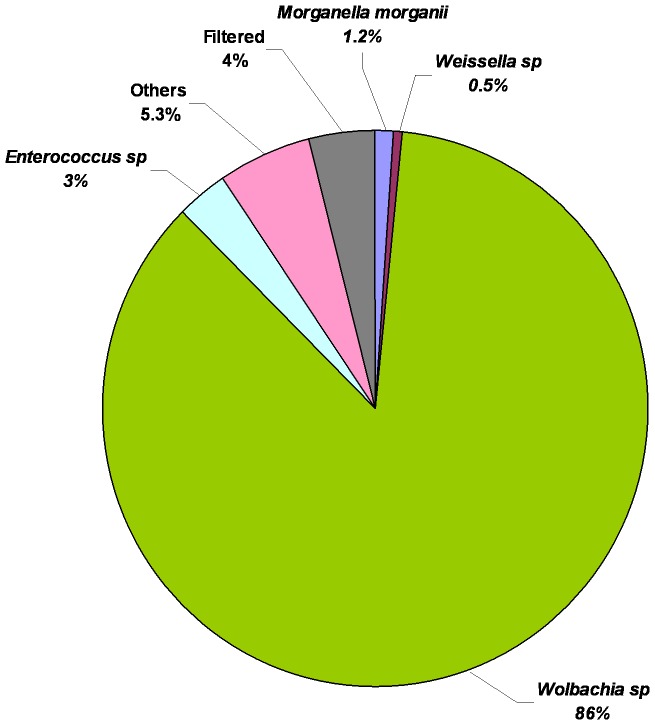
Relative abundance of bacterial genera/species in horn fly eggs after applying the cut-off filter of abundance level ≥0.1% and prevalence of taxa ≥66% within each class respectively or a prevalence ≥55% across all nine samples. Values are mean percentages (n = 3).

Among the 28 OTUs identified in horn fly eggs *Clostridium lituseburense*, *Morganella morganii*, *Staphylococcus saprophyticus*, *Serratia marcescens*, *Staphylococcus hyicus* and *Staphylococcus sciuri* were the bacteria identified to the level of species. *Wolbachia* was the most abundant (86%) bacteria in fresh horn fly egg. With the exception of *Enterococcus* (3%) and *Morganella morganii* (1.2%), all other OTUs were less than 1% abundance (Fig. 6).

### Comparison of bacteriomes via phylogenetic information

**Figure 7:**
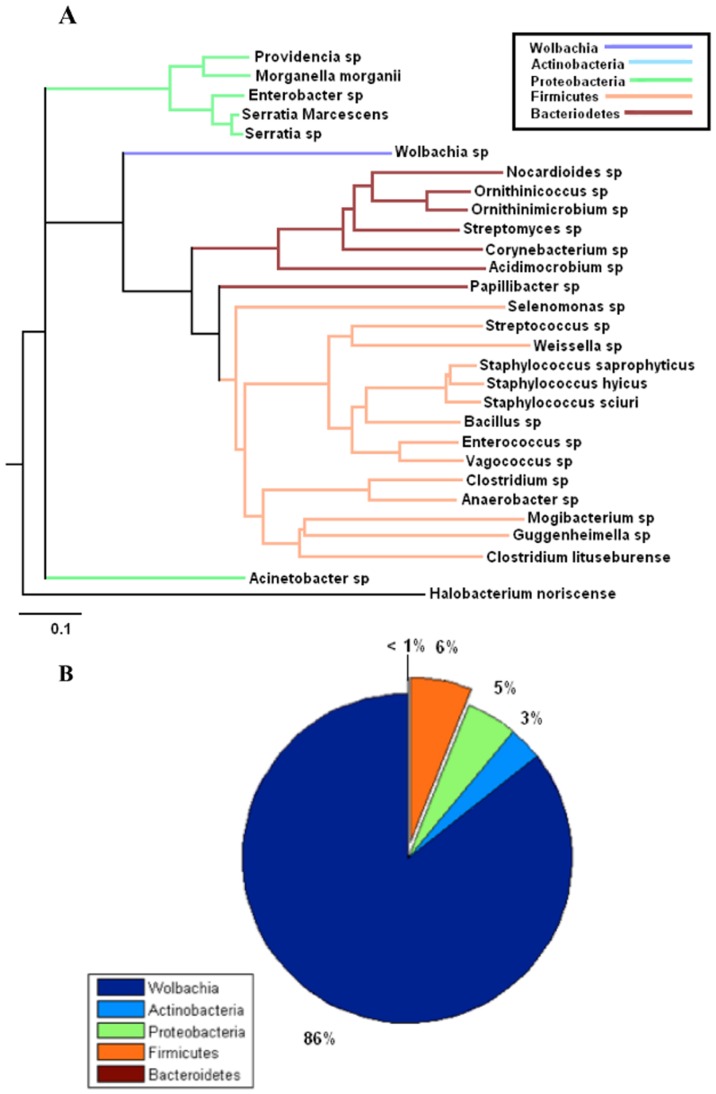
Selected 16S rDNA sequences from the horn fly egg bacteriome. **a**. Phylogenetic tree of selected 16S rDNA sequences from horn fly egg bacteriome based on cut-off filter of abundance level ≥0.1% and prevalence of taxa ≥66% within each class respectively or a prevalence ≥55% across all nine samples. The scale bar represents 0.1 changes per nucleotide position. *Halobacterium noricense* was used as the outgroup. b. The percent relative abundance of the major phyla found within the 16S rDNA sequences from the horn fly egg bacteriome. The relative abundance of *Wolbachia* is included for comparison.

**Figure 8:**
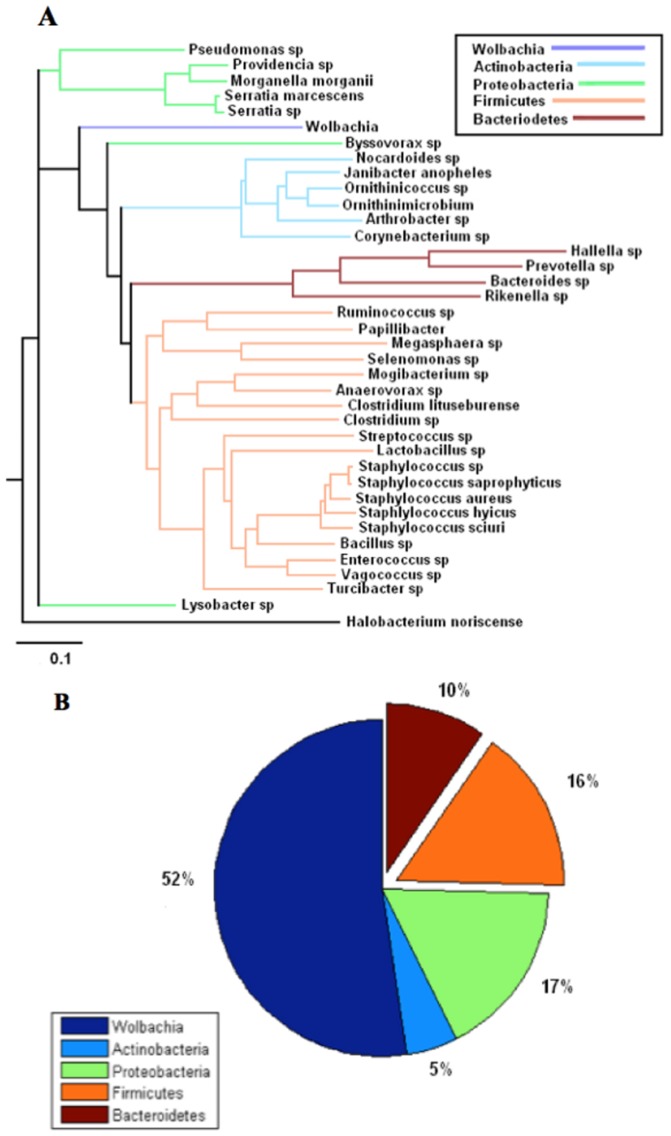
Selected 16S rDNA sequences from the male horn fly bacteriome. **a**. Phylogenetic tree of selected 16S rDNA sequences from the male horn fly bacteriome based on cut-off filter of abundance level ≥0.1% and prevalence of taxa ≥66% within each class respectively or a prevalence ≥55% across all nine samples. The scale bar represents 0.1 changes per nucleotide position. *Halobacterium noricense* was used as the outgroup. b. The percent relative abundance of the major phyla found within the 16S rDNA sequences from the male horn fly bacteriome. The relative abundance of *Wolbachia* is included for comparison.

**Figure 9:**
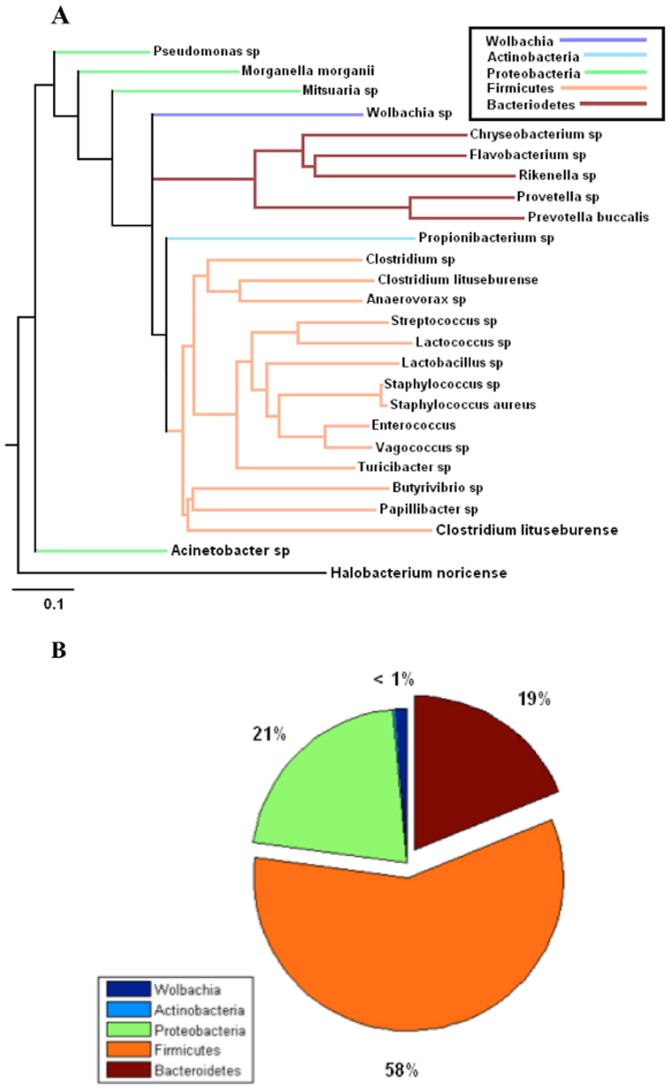
Selected 16S rDNA sequences from the female horn fly bacteriome. **a**. Phylogenetic tree of selected 16S rDNA sequences from female horn fly bacteriome based on cut-off filter of abundance level ≥0.1% and prevalence of taxa ≥66% within each class respectively or a prevalence ≥55% across all nine samples. The scale bar represents 0.1 changes per nucleotide position. *Halobacterium noricense* was used as the outgroup. b. The percent relative abundance of the major phyla found within the 16S rDNA sequences from the female horn fly bacteriome. The relative abundance of *Wolbachia* is included for comparison.

Trees signifying evolutionary distance for the male, female, and egg bacteriomes were generated, respectively, with the Phylodendron phylogenetic tree printer (Figures 7a, 8a, and 9a) [27]. Pie charts indicating the relative abundance of phyla present in each bacteriome are shown in figures 7b, 8b, and 9b. Clades representing different phyla are indicated by color. Note that *Wolbachia*, though it is a member of the proteobacteria phylum, was represented as a separate taxon and hence given its own color to highlight its relationship to the other OTUs. Taken together, these figures demonstrate how the relative abundance of each phylum is related to its evolutionary distance from *Wolbachia* in all three environments.

Performing the P test, which ignores abundance information and takes only phylogenetic information into account, the horn fly egg bacteriome was found to be significantly different than the female horn fly bacteriome (*p* = 0.002). Also, the male and female bacteriomes were significantly different (*p* = 0.002). Statistically speaking the male and egg bacteriomes were similar (*p* = 0.056). However, these tests imply a qualitative difference between the female horn fly and the horn fly egg bacteriomes. The male bacteriome appears to be intermediate between the female and egg, lying closer to the egg microbiome. This was also reflected in the heat map shown in Figure 1.

## Discussion

Identifying and characterizing the microflora associated with arthropod pests of livestock offer the prospect for new avenues to control the insect population and the reduction in the vectorial capacity of the arthropod vector. Research on the identification of primary obligatory symbionts of insects may enable novel approaches to control insect borne diseases or insect populations through stable insect paratransgenesis [28]. Insect paratransgenesis is an approach whereby an insect symbiont is transformed to express effector molecules inside the insect to kill the pathogen/parasite transmitted by the insect or to express a lethal protein inside the insect to control the insect population. *Rhodococcus rhodnii*, a symbiont of the reduviid bug *Rhodnius prolixus* has been identified as a potential symbiont to control transmission of *Trypanosoma cruzi* infection using the paratrangenesis approach [29]. Similarly, *Sodalis glossinidius*, a symbiont of tsetse flies and *Asaia* sp., an acetic acid bacterial symbiont of *Anopheles stephensi* have the potential to be used in paratransgenic approaches to control African trypanosomiasis and malaria [30]–[33]. Insects feeding exclusively on a particular food source require the help of symbionts to supplement nutrients for their normal growth and development. For example, *Buchnera aphidicola*, a symbiont of aphids, synthesizes and supplies amino acids to aphids [34]. The horn fly, which exclusively feeds on animal blood, could be an interesting insect model to study the nutritional interaction between symbionts and their insect hosts.

This is the first comprehensive report that exploring the diversity and abundance of bacterial genera/species associated with the horn fly using non-culture-based approaches. Two previous studies used conventional bacterial culture methodology to report on bacterial communities associated with Canadian populations of the horn fly. *Achromobacter, Aerobacter aerogenes*, *Alcaligenes*, *Bacillus subtilis*, *B. subtilis var.niger*, *Escherichia coli*, *Micrococcus candidus*, *M. caseolyticus*, *M. epidermidis*, *M. flavus*, and *Providencia rettgeri* were isolated from horn flies collected from Lethbridge, Canada [35]. Another research group from Canada reported *Bacillus licheniformis*, *B. pumilus*, *Escherichia coli*, *Pantoea agglomerans*, *Providencia rettgeri*, *Acinetobacter* and *Comamonas acidovorans* from the gut of horn fly larva [36]. They also found that *P. rettgeri* was the most abundant bacterial species in the larval gut followed by *B. licheniformis*, *B. pumilus* and *P. agglomerans*. In the present study, we identified 50 abundant bacterial genera/species from adult males, adult females, and eggs, including *Bacillus*, *Providencia*, and *Acinetobacter*, genera that were identified in the Canadian studies. *Achromobacter*, *Aerobacter*, *Alcaligenes*, *E. coli*, *Micrococcus*, *Pantoea* and *Comamonas acidovorans* were the bacteria reported to be present in the Lethbridge laboratory colony flies that were not detected in our fly samples. The difference in the horn fly microflora of US and Canada isolates could be attributed to the difference in geographical location, season and sensitivity of detection techniques [37]. Another Sensitivity of the methodology used to detect the bacteria is an important factor that influences the quality of studies on insect microflora. More than 99% of microorganisms existing in nature cannot be isolated and maintained in pure culture [19]. Thus, culture-based methods will be severely limited in the breadth of species and genera that can be identified and should be supplemented with molecular-based approaches to gain a more thorough understanding of the microbiome of an organism.

Adult male and female horn flies are obligatory blood feeders on cattle, although adult flies have occasionally been observed drinking the fluid on the surface of freshly passed cattle manure [38]. The larval stage lives only in fresh cattle manure. Bacteria are carried over in small numbers from the larval to the pupal and adult stages of the horn fly [39]. Horn fly larvae failed to develop in sterilized, uninoculated manure, indicating that manure bacteria are necessary for larval development [36]. These observations suggested that the horn flies might obtain necessary nutrients from microorganisms in their environment. The association of many rumen, gastero-intestinal, skin and environmental bacteria of animals with the horn fly in our study confirms that this economically important insect species acquires bacteria from manure, animal body and soil (Table 3).

**Table 3:** Rumen, environmental and pathogenic bacteria associated with horn fly (*Haematobia irritans*).

Rumen/Gastro-intestinal bacteria of animals*	Natural skin and oro-nasal flora of animals/environmental bacteria	Pathogenic/opportunisticPathogen
*Lysobacter sp*	*Mogibacterium sp*	*Morganella morganii*
*Acinetobacter sp*	*Hallella sp*	*Staphylococcus aureus*
*Ruminococcus sp*	*Chrysebacterium sp*	*Staphylococcus saprophyticus*
*Clostridium sp*	*Anaerobacter sp*	*Staphylococcus hyicus*
*Megasphaera sp*	*Streptococcus sp*	*Serratia marcescens*
*Butyrivibrio sp*	*Corynebacterium sp*	
*Lactobacillus sp*	*Guggenheimella sp*	
*Providencia sp*	*Prevotella buccalis*	
*Turicibacter sp*	*Staphylococcus sp*	
*Papillibacter sp*	*Flavobacterium sp*	
*Prevotella sp*	*Pseudomonas sp*	
*Selenomonas sp*	*Clostridium lituseburense*	
*Enterococcus sp*	*Morganella morganii*	
*Bacteroides sp*	*Mitsuaria sp*	
	*Staphylococcus aureus*	
	*Arthrobacter sp*	
	*Ornithinicoccus sp*	
	*Propionibacterium sp*	
	*Serratia sp*	
	*Serratia marcescens*	

* See reference number 26.

Several environmental bacteria including skin and oro-nasal bacteria of animals were associated with the horn fly (Table 3). Among these, *Pseudomonas*, *Staphylococcus*, *Clostridium lituseburense* and *Morganella morganii* were detected in adult males, adult females, and eggs. *Chryseobacterium* sp., a soil and aquatic bacteria which was also found in the gut of the American cockroach, was detected in adult female horn fly. The *Chryseobacterium* sp. isolated from American cockroach was phylogenetically and phenotypically similar to *C. indologenes* and thought to be a symbiont of American cockroach [40]. *Flavobacterium*, a bacterium abundant in soil and water, was detected in female horn flies and this bacterium is well known for its involvement in biotransformation of organophosphate pesticides. *Mitsuaria*, a chitosanase-producing soil bacterium, was detected in adult female flies. Chitosan is a deacetylated derivative of chitin used as eco-friendly bio-pesticide [41]. Chitosan was also found in house fly larvae [42]. Presence of chitosanase-producing bacteria suggests the involvement in degrading chitosan of horn fly life stages during their development. *Arthrobacter*, another soil bacterium detected in adult male horn fly has the ability to reduce hexavalent chromium and degrade the organophosphate diazinon [43]. *Serratia* are environment bacteria detected in adult males and eggs and are well known for the production of a reddish-orange pigment, prodigiosin, which has antimicrobial properties [44], [45]. Acknowledging the physical presence of all these environmental bacteria in horn fly does not necessarily indicate a biological relationship exists between the horn fly and bacteria. The nature of these relationships between the identified bacteria with the horn fly, whether they are simple physical associations, symbiotic, or commensal needs further investigation. However, keeping in mind the biochemical properties of these bacteria and their association with horn fly, some of these bacteria could have established a commensal or symbiont relationship with horn fly.

Several pathogenic bacteria were found associated with the horn fly, including *Staphylococcus aureus*, *S. saprophyticus*, *Morganella morganii* and *Serratia marcescens*. The pathogenic bacteria of horn fly detected in this study could have been acquired from pastured cattle. *S. aureus* is part of the normal animal skin flora and horn flies have been shown to be capable of transmitting *S. aureus*-induced mastitis in bovines [13], [46]. *S. aureus* was found both in adult males (0.6% abundance) and females (22% abundance) and was the most abundant bacteria in adult female horn fly. This result confirms the earlier reports of horn fly colonization by *S. aureus* [13] and suggests that female horn flies have a higher vectorial capacity to transmit *S. aureus* than male flies. Interestingly, our study found an inverse relationship between the abundance of *Wolbachia* and that of *S. aureus* in adult female and male flies. The abundance levels for *S. aureus* and *Wolbachia* in the adult female sample was 22% and 1%, while the adult male sample had 0.6% and 52.4%, respectively. Vectorial capacity of an insect vector is influenced by *Wolbachia* infection, as *Wolbachia* is known to inhibit virus replication inside insect vectors [47], [48]. *Wolbachia* also increases the antioxidant and reactive oxygen species level in insects [49]. Some *Wolbachia* strains reduce the life span of insects, which ultimately impacts their vectorial capacity [50]. Perhaps *Wolbachia* regulates the colonization of *S. aureus* in horn fly and alters the vectorial capacity of the horn fly to transmit *S. aureus*. *S. saprophyticus* is a common meat contaminant and implicated in urinary tract infection of man [51], [52] and was found in the adult male horn fly and horn fly egg. *Morganella morganii* was detected in low abundance in females (0.5%), males (0.4%), and eggs (1.2%). It is an environmental bacterium most often encountered in post-operative and nosocomial infections and is also implicated as a causative agent of summer diarrhea in humans [53]. *Serratia marcescens* is a ubiquitous environmental bacterium involved in nosocomial and urinary tract infections and was detected in adult male horn fly (0.6%) and eggs (0.1%) in low abundance [54]. *Vagococcus* was found in the adult female, adult male, and egg samples in low abundance. The association of *Vagococcus* with the *Lucilia sericata* maggot and its resistance to the excretory-secretory products of the maggots indicates a possible symbiotic relationship with *L. sericata*. It is possible the horn fly and *Vagococcus* have also established a symbiotic relationship [55].

*Rikenella* was detected in adult females and males in moderate abundance, 8.1% and 2.9%, respectively. *Rikenella* is a symbiont of the medicinal leech, *Hirudo verbana*, where this bacterium dominates the crop microbiota along with another bacteria *Aeromonas veronii* [56]. *Rikenella* is an obligatory anaerobic bacterium that seems to help the leech to digest the blood in the crop. Evidence suggests the involvement of *Rikenella* bacteria in fermentation of mucin glycans to acetate, which may serve as an energy source for leech enterocytes [57], [58]. Both the horn fly and leech are obligatory blood feeders, exclusively living on a blood diet. *Rikenella* have been detected in low abundance (0.1%) in adult male cattle ticks, *Rhipicephalus microplus* [59], another obligate blood feeder. Interestingly, *Rickenella* has not been reported in mosquitos, which feed on both blood and nectar, leading us to speculate that *Rikenella* is a symbiont evolved to co-exist with obligatory blood feeding pests. The presence and abundance of *Rikenella* in horn fly, especially its relatively high abundance in the female horn fly (8.1%) supports the concept of a symbiotic relationship. Female horn flies ingest a much higher quantity of blood than males and the higher abundance of *Rikenella* in females compared to males could be correlated with the amount of blood taken. Unfortunately, the *Rikenella* symbiont of the leech remained uncultured despite several isolation attempts and it would be difficult to confirm the biological relationship of this bacterium with horn fly by culture-based methods [58].

*Wolbachia* are intracellular bacteria naturally present in large number of insects and other arthropod species. They are maternally transmitted across generations through eggs and confer reproductive advantage to infected females through cytoplasmic incompatibility, feminization, male killing or parthenogenesis [60]. *Wolbachia* were found in all three life stage samples, and it was the most abundant bacteria found in adult males (52.4%) and eggs (86%). The abundance level of *Wolbachia* in adult females was only 1%. This comparatively low value might be explained by our experimental design and sampling method. Adult female flies were collected and incubated for 1.5 hrs in the dark to induce oviposition. Following this period of oviposition, the adult flies and eggs were collected and immediately frozen for preservation of DNA. If the female flies had oviposited all their eggs, there would be little time for the next round of oogenesis and subsequent self-renewal, amplification within the ovaries, and transfer of *Wolbachia* to the next generation of oocytes. These results also suggest that the *Wolbachia* in horn fly multiplies inside the eggs and not in the adult female fly ovary or body tissues, which is in agreement with *Wolbachia* biology in *Drosophila* [61], [62]. There is evidence suggesting that the replication of *Wolbachia* is dependent on host cell replication [63]. The rapid cell division at the early stage of embryogenesis could be attributed to the high abundance of *Wolbachia* in eggs. This is the first report of *Wolbachia* in horn flies from the USA. *Wolbachia* has been reported from Canadian, Mexican and European horn fly populations [64]–[67]. *Wolbachia* has not been reported in the buffalo fly, *Haematobia irritans exigua*, a close relative of the horn fly that is of Australia and Indonesia origins [68]. Furthermore, the presence of *Wolbachia* in horn flies of Canada, Mexico, Europe and USA reinforce the differences between the horn fly of North America and Europe and the buffalo fly of Australia and Indonesia, perhaps to the extent that the presence of *Wolbachia* is the characteristic that drove the speciation between *H. irritans irritans* and *H. irritans exigua*. The horn fly is believed to have been introduced into the USA from France prior to 1887 [69]. The buffalo fly, *Haematobia irritans exigua*, was introduced into Northern Australia in 1838 from Indonesia [70]. The presence and absence of *Wolbachia* in the horn fly and buffalo fly, respectively, could perhaps ultimately be attributed to continental drift theory, as North America and Europe were part of Laurasia while Australia, India, and Indonesia were part of Gondwanaland [71]. Investigations on the presence of *Wolbachia* in buffalo fly and horn fly population of Asia, particularly the Indian subcontinent may provide interesting findings on the divergent evolution of these two closely related fly species.

The phylogenetic analysis revealed that horn fly eggs have a significantly different composition of their microbiota when compared with adult male and female horn flies. This difference is due primarily to a substantial increase in the relative abundance of the *Wolbachia* when compared to the adult fly microbiota. However, we acknowledge that the different levels of sampling depth of the male samples when compared with the sampling depth of the female and egg samples limits the ability to make direct comparisons of the male horn fly microbiome with the female and egg microbiomes. Figures 7b, 8b, and 9b show a decrease in OTUs in the Firmicutes phylum, the predominant gram positive group present in the data, from over 58% in adult females to almost 16% in adult males and only 6% in horn fly eggs, respectively. The abundance of Bacteroidetes, the predominant gram negative phylum present in the data that does not include *Wolbachia*, likewise decreased from 18% in adult females to about 9% in adult males and were nearly depleted in horn fly eggs. As indicated in the phylogenetic trees from the same figures, the Firmicutes and Bacteroidetes OTUs tend to be the most distantly related taxa to *Wolbachia*. This negative correlation between the abundances of Firmicutes and Bacteroidetes and the abundance of *Wolbachia* may be due to an as yet unknown mechanism that favors *Wolbachia* and related taxa to the detriment of less related taxa. This dramatic shift in composition of the host microbiota may also indicate a gain in functional diversity in the adult microbiomes when compared with the egg microbiome. As more diverse taxa appear in the community, the functional capacity of that microbiome increases [72]. The lack of diversity in the egg microbiome is supporting evidence of the singular role that *Wolbachia* plays in horn fly reproduction.

In the present study, diversity and abundance of bacterial microflora of the horn fly were profiled using pyrosequencing and bioinformatic analysis of the sequences. Fifty bacterial genera and species were shortlisted from a total of 363 genera/species based on the abundance and prevalence across samples. Though we report the abundance of bacterial communities associated with horn fly, it is critical to acknowledge the potential for mismatch of actual species abundances in a community and sequence abundances in DNA samples due to number of factors including storage conditions of samples, sequencing of dead cells and PCR biases with the primers used. Horn fly associated rumen bacteria, environmental bacteria, pathogenic bacteria and possible symbiotic bacteria were profiled based on the available literature. This is the first report of the presence of *Wolbachia* in horn flies from the USA and also the first report of presence of *Rikenella* in an obligatory blood feeding insect. Although the present study reports the qualitative and quantitative survey of the bacterial flora associated with the horn fly, the isolation and functional characterization of the bacterial species needs to be done to understand the role of these bacteria in horn fly biology.

## Materials and Methods

### Horn fly

Adult horn flies were collected on a single date from pastured cattle at the Louisiana State University Agricultural Center, St. Gabriel Research Station using aerial nets. Within 1 h after collection the flies were transferred to large sterile Erlenmeyer flasks and maintained in total darkness for 1.5 h and 30°C to allow flies to oviposit on the flask bottom [73]. Adult flies were released from the flasks into a cage and eggs were collected by rinsing with distilled water onto a filter paper. Both the eggs and adult flies were frozen at −80°C. To preserve nucleic acid integrity, adults were sexed on dry ice prior to freezing. Each sample used for DNA extraction and pyrosequencing consisted of 5 adult males, 5 adult females or 50 eggs pooled together and homogenized. Three replicates of adult male, adult female and eggs were analyzed.

### Pyrosequencing and Statistical Approach

Previously described methods for bTEFAP and data processing were utilized with some modifications [21], [22]. The bTEFAP was based upon the titanium sequencing platform rather than FLX (Roche Applied Science, Indianapolis, IN). The titanium sequencing differs from the FLX in that it generates average read lengths of 400 bp rather than 250 bp as with FLX. Also different from the previous studies were the primers utilized, which extended from 27F numbered in relation to the *Escherichia coli* 16S ribosome gene. Finally, rather than the two step PCR protocol utilized previously, only a single step reaction with 30 cycles was used and 0.5 U of HotStar HiFidelity Polymerase (Qiagen Inc, Valencia, CA) was added to each reaction. Raw data from bTEFAP was screened and trimmed based upon quality scores and binned into individual sample collections. Sequence collections were depleted of chimeras using B2C2 (www.researchandtesting.com/B2C2). The resulting files were then depleted of short reads (<350 bp), reads with ambiguous base calls, reads with homopolymers >6 bp, and reads with lower than Q25 quality scores. Bacterial classifications were performed using BLASTn comparison to a curated high quality 16S database (Research and Testing Laboratory, Lubbock, TX). Data were compiled and relative abundances of bacteria were determined for each individual sample, using abundance as an ecological concept referring to the relative representation of a species in a particular ecosystem. Data were also compiled at each taxonomic level according to the NCBI taxonomy criteria as described previously [21], [22]. Principle component analysis was conducted and double dendrogram heat maps were derived as described in [74].

### Phylogenetic analysis of the horn fly bacteriome

A multiple sequence alignment (MSA) was performed using the PyNAST alignment tool on the sequence data meeting the inclusion criteria of >1% abundance [75]. The data were aligned with a template of previously aligned 16S rDNA sequences. The template database, Greengenes, is a curated database of over 117,000 16S rDNA aligned sequences with taxonomic assignments [76]. Initially a representative 16S read from each Operational Taxonomic Unit (OTU) was randomly chosen. A BLAST search using the representative read as the query string was then performed. We identified the full 16S rDNA sequence for each read from the best BLAST search hit to the Greengenes database. These full 16S rDNA sequences were subsequently aligned with the template database.

Two applications from the PHYLIP package, NEIGHBOR and DNADIST, were used to prepare the abundance data for rendering as phylogenetic trees [77]. DNADIST computes a distance matrix of pairwise distances between all sequences in the MSA under the Jukes and Cantor model [78], where all sites within a nucleotide sequence are assumed to change independently with equal probability. The resulting distance matrix serves as the input to the clustering step. Using the UPGMA option, the NEIGHBOR application was used to construct a tree via agglomerative clustering using an average-linkage method.

The online tool UniFrac was employed to make a pair-wise comparison of male, female and horn fly egg bacteriomes [79]. UniFrac provides a measure of community composition in a phylogenetic context to test the null hypothesis that two samples were drawn from the same community versus the alternative hypothesis that the samples were drawn from different communities. From the tree generated by the previous clustering step, a *P* test was performed by Unifrac that estimated similarity between microbiome environments as the number of parsimony changes (change from one sample to another along a given branch) that would be required to explain the distribution of sequences between the different samples in the tree [80]. Tree permutations were used to generate a random distribution of 500 trees to which the true tree was compared. The *P*-value is the proportion of trials in which the true tree requires fewer changes than the trees in which the environment labels were randomly chosen. All *P*-values were corrected for multiple comparisons (Bonferroni correction).

## Supporting Information

Table S1 **This file is a table in excel format.** The file contains bacterial species load in the adult female horn flies, the adult male horn flies and horn fly eggs. Bacterial load was determined by dividing the 16S rDNA numbers representing the tally for each species by the total tally detected in each life stage. We report the average from the three replicates in the text.
(XLS)

Figure S1 **Rarefaction curves plotted at 0.03 divergence level for all nine samples of horn fly.** The rarefaction curves imply a depth of coverage of approximately 10000 sequences/sample for the female and egg samples. The male horn fly samples were only sampled to about one third of the depths of either the female or egg samples.
(JPG)
