# Supplementary figures and images for: Pyrosequencing-Based Analysis of the Microbiome Associated with the Horn Fly, Haematobia irritans
Source: PLoS One. 2012 Sep 24;7(9):e44390. doi: 10.1371/journal.pone.0044390 (PMC3454415; doi:10.1371/journal.pone.0044390)

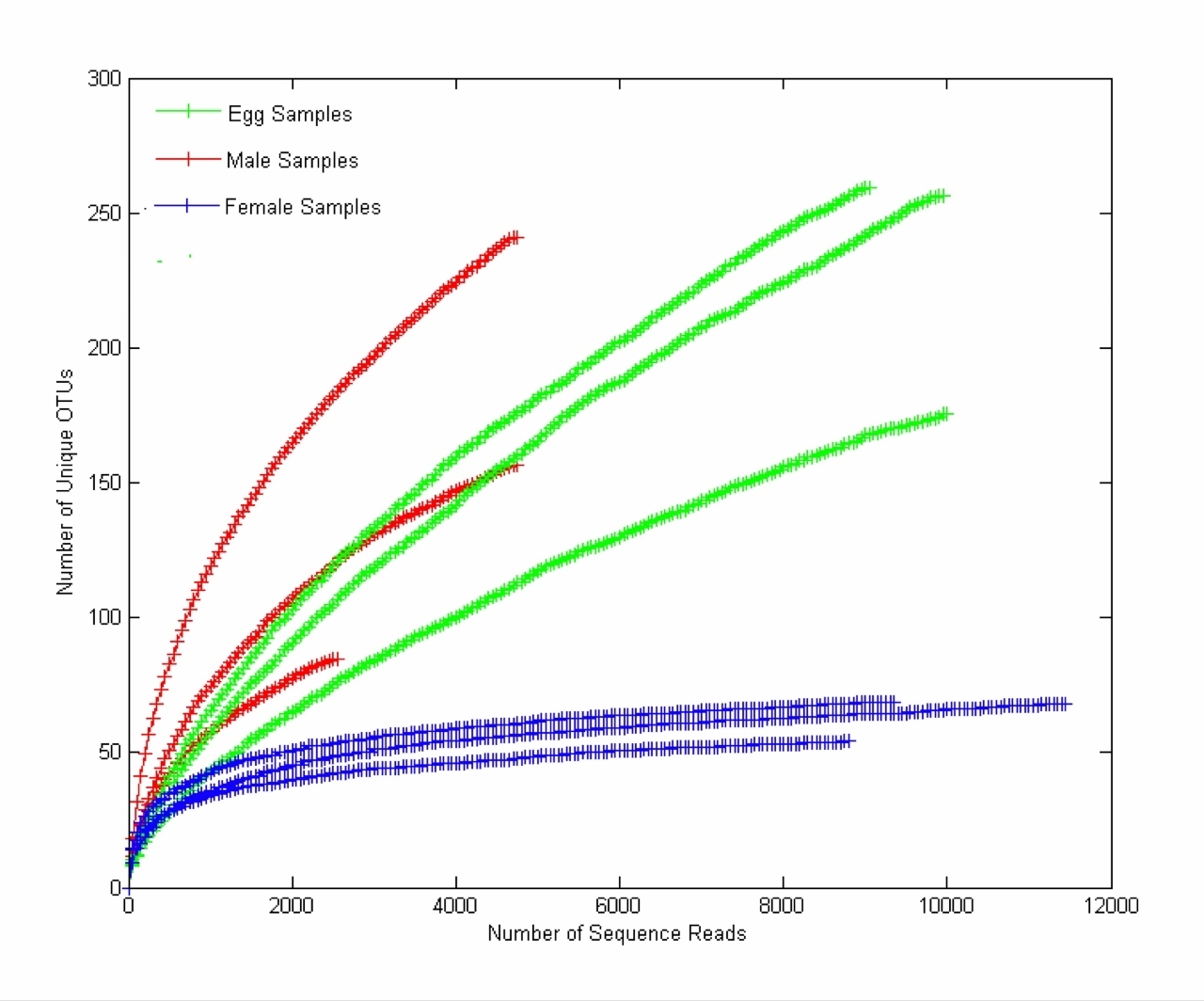

Supplement: Figure S1 — Rarefaction curves plotted at 0.03 divergence level for all nine samples of horn fly. The rarefaction curves imply a depth of coverage of approximately 10000 sequences/sample for the female and egg samples. The male horn fly samples were only sampled to about one third of the depths of either the female or egg samples. (JPG) [file pone.0044390.s002.jpg]
